# A modified, single-incision, gasless, endoscopic thyroidectomy and bilateral central neck dissection via axillary approach technique for bilateral papillary thyroid microcarcinoma: A preliminary report

**DOI:** 10.1016/j.heliyon.2024.e24802

**Published:** 2024-01-23

**Authors:** Shi-Tong Yu, Jun-Na Ge, Bai-Hui Sun, Zhi-Gang Wei, Zhi-Cheng Zhang, Wei-Sheng Chen, Ting-Ting Li, Shang-Tong Lei

**Affiliations:** Department of General Surgery, Nanfang Hospital, The First School of Clinical Medicine, Southern Medical University, Guangzhou, Guangdong Province, China

**Keywords:** Endoscopic thyroidectomy, Thyroid cancer, Transaxillary endoscopic surgery

## Abstract

**Background:**

Our objective was to assess the viability and oncological security of a gasless, transaxillary single-incision endoscopic procedure for performing total thyroidectomy and bilateral central neck dissection (TT + BCND). This study focused on patients diagnosed with bilateral papillary thyroid microcarcinoma (PTMC).

**Method:**

Between April 2020 and November 2021, 22 patients with bilateral PTMC underwent single-incision, gasless, transaxillary endoscopic TT + BCND. The patients’ clinicopathologic characteristics, surgical completeness and complications were analyzed.

**Result:**

Single-incision, gasless, transaxillary endoscopic TT + BCND was successful performed in all patients. The median (IQR) total surgical time was 143 (85–160) min. Only two patients experienced transient unilateral RLN palsy or transient hypocalcemia. All these complications resolved within 1 month after surgery. The median duration of hospital stay after surgery was 4 (3–4.5) days. The median hospitalization expense for these patients was 3848 (3781–4145) USD. The median number of lymph node yielded was 10.5 (8–15). The cosmetic outcomes were well-received by all individuals.

**Conclusion:**

In certain cases, gasless, transaxillary endoscopic TT + BCND procedure performed through a single incision proved to be a secure alternative for managing bilateral PTMC.

## 1 Introduction

The incidence of thyroid cancer, the most prevalent malignancy in the endocrine system, has increased worldwide [1,2]. Due to the improvement in the sensitivity of ultrasonography, papillary thyroid microcarcinoma (size ≤1 cm, PTMC) accounts for the majority of newly diagnosed cases [3]. To achieve better cosmetic and oncological results, remote access techniques, including the transaxillary approach, have been introduced in thyroid surgery [4]. Since Ikeda et al. reported the first endoscopic transaxillary thyroidectomy in 2000 [5], this technique has gained widespread acceptance due to its numerous advantages: a surgical view akin to open surgery, an expansive field for operation, and the ability to access the thyrothymic ligament and perform central compartment neck dissection [6]. However, this approach does not allow easy visualization of the contralateral thyroid or the lymph nodes in the central neck since these are located at the blind angle and are blocked by the trachea. Therefore, some studies have reported an endoscopic or robot-assisted unilateral transaxillary approach for bilateral thyroidectomy and unilateral central neck dissection [[6], [7], [8]].

Based on our previous studies [9,10], we described a posterior approach for endoscopic unilateral thyroidectomy and central neck dissection. To expand its indication to bilateral lesions, especially those that need bilateral central neck dissection, we proposed a modified surgical technique, making endoscopic total thyroidectomy (TT) and bilateral central neck dissection (BCND) a more feasible method. Therefore, the goal of this study was to report the technique details with a relatively small cohort and evaluate the viability and oncological security of single-incision, gasless, transaxillary endoscopic TT + BCND in patients with bilateral PTMC.

## 2 Materials and methods

### 2.1 Study design

At the Department of General Surgery, Nanfang Hospital, Southern Medical University, single-incision, gasless, transaxillary endoscopic TT + BCND surgery was performed on 22 patients with bilateral PTMC between April 2020 and November 2021. This study strictly followed the recommendations stated in the STROBE statement with regards to its design, development, analysis, and reporting.

The inclusion criteria were as follows: (1) patients with bilateral PTMC confirmed by preoperative ultrasound-guided fine needle aspiration cytology or highly suspicious ultrasonography features; (2) patients who refused open surgery or bilateral axillary incisions; and (3) patients’ selection. The exclusion criteria were as follows: (1) PTMC patients with lateral neck metastases (cN1b), or distant metastases (M1), or cT3 or T4 at the preoperative assessment; and (2) patients with a previous history of surgery or irradiation on the neck. One experienced surgeon (L-ST) performed all operation. According to the Chinese guidelines, prophylactic bilateral CND was performed for all patients. All of the patients received levothyroxine, a hormone replacement, after thyroidectomy and thyroid stimulating hormone (TSH) suppression therapy. Tg/TgAb and ultrasonographic images were examined one month, 6 months, and one year after surgery.

### 2.2 Operative techniques

**Fig. 1:**
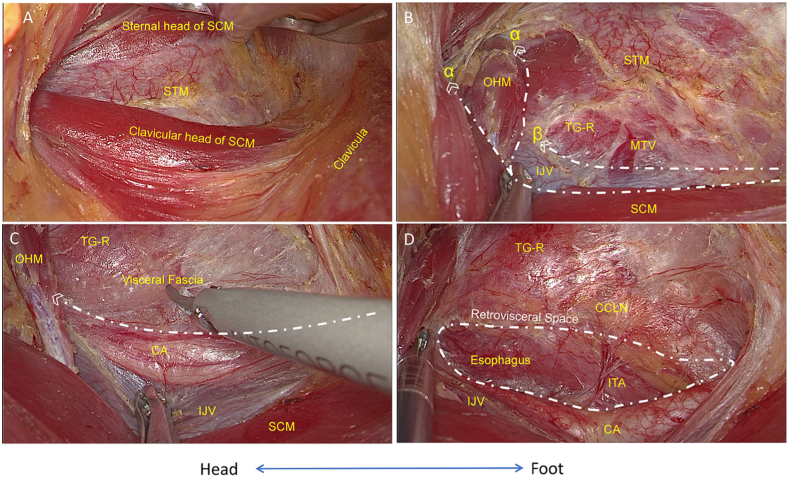
**Surgical plate creation.** A) The clavicular and sternal heads of the SCM muscle. B) Line α shows that the STM was dissected laterally to expose the OHM and IJV, and line β shows that the carotid sheath was exposed along the IJV. C) The inner side of the carotid sheath and dissection of the visceral fascia longitudinally. D) The posterior boundaries of the thyroid gland and the CCLN (surgical plate) were exposed by revealing the retrovisceral space and esophagus. Note: SCM, sternocleidomastoid muscle; STM, sternothyroid muscle; OHM, omohyoid muscle; IJV, internal jugular vein; MTV, middle thyroid vein; TG-R, right thyroid gland; CA, carotid artery; ITA, inferior thyroid artery; CCLN, central compartment lymph nodes. White lines represent cutting lines.

The procedure of endoscopic TT + BCND was similar to endoscopic thyroid lobectomy, as we described [9,10]. The main element of technique is to use a specially designed retractor to pull the thyroid and surrounding tissue upwardly. The posterior aspect of the thyroid can be revealed and therefore the dissection can be made from a posterior approach. Specifically, after general anesthesia, the patient was placed in a supine position with the neck extented. The right arm was extended 90°**.** Initially, a vertical incision measuring 5 cm was conducted, commencing from the line in front of the axilla and extending along the natural creases in that region. Following this, the superficial fascia of the pectoralis major muscle was meticulously dissected with a electrical cautery, ensuring direct vision, until the sternocleidomastoid muscle (SCM) became visible. Subsequently, an entry point for 5 mm trocar, was established at the indentation caused by the upper lateral quadrant of the breast tissue and the line in front of the axilla, at a distance of 5 cm away from the incision. Eventually, a 30-degree endoscope was carefully used through the incision in the axillary area. After the clavicular and sternal heads of the SCM muscle were exposed by dissecting the SCM longitudinally, the specially designed retractor (Yuan Xing, co Ltd. Shanghai, China) was placed to expose the sternothyroid muscle (STM). Then, the STM was dissected laterally to expose the omohyoid muscle and the carotid sheath. Accordingly, the working space was created by the retractor (Fig. 1A–C).

Under endoscopic guidance, a harmonic scalpel (Johnson & Johnson, Cincinnati, USA) was used to expose the inner side of the carotid sheath and dissect the visceral fascia longitudinally. First, the carotid artery was traced to identify and ligate the inferior thyroid artery (ITA). Simultaneously, the inferior parathyroid glands (PTGs) were identified and preserved, along with their blood supplies derived from the ITA. Next, the retrovisceral space (superficial space to the prevertebral fascia) was identified and dissected from the sternal notch toward the thyroid therefore reveal the esophagus. The posterior boundaries of the thyroid and CCLN were exposed (Fig. 1D).

**Fig. 2:**
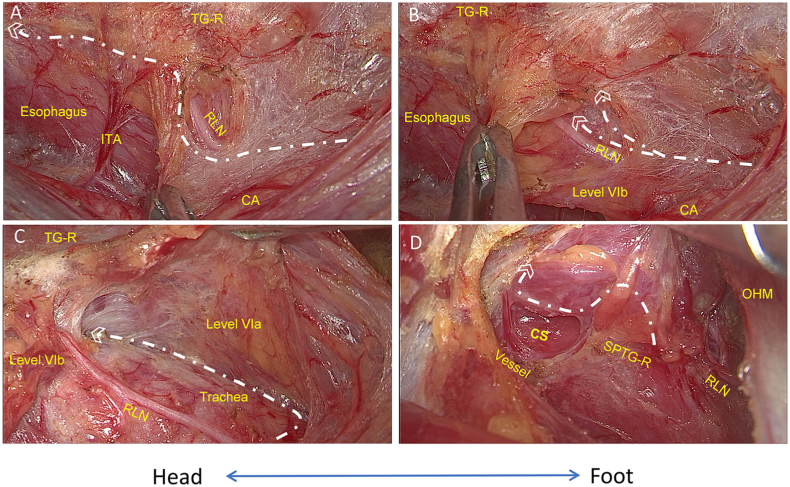
**Management of the right thyroid gland and pretracheal space.** A) The right RLN was identified at the starting point (inside of the carotid artery). B) Level VIb was separated by dissecting the buccopharyngeal fascia. C) The right RLN was traced to the ending point (entering the larynx), while the pretracheal space was expanded to reveal level VIa. D) The superior vessels of the TG-R were ligated by exposing the cricothyroid space while the SPTG-R was preserved. Note: ITA, inferior thyroid artery; TG-R, right thyroid gland; RLN, recurrent laryngeal nerve; CA, carotid artery. OHM, omohyoid muscle; SPTG-R, right superior parathyroid gland; CS, cricothyroid space. White lines represent cutting lines.

Moreover, upon dissection of the buccopharyngeal fascia, the esophagus was isolated in a vertical orientation, commencing from inferior to superior. Subsequently, the right recurrent laryngeal nerve (RLN) was identified and detached from the superomedial aspect of the brachiocephalic trunk. This path was traced intraoperatively using neuromonitoring techniques (Medtronic Xomed Inc., Jacksonville, USA), commencing at the initial spot within the carotid artery and culminating upon entry into the larynx. Additionally, Level VIB lymph nodes were excised during the procedure. (Fig. 2A–C). Using the harmonic scalpel, the superior thyroid vessels were individually ligated. Meanwhile, we properly preserved the external branch of the superior laryngeal nerve (EBSLN) and superior PTGs (Fig. 2D). Then, starting from exposing the cricothyroid space, the prelaryngeal lymph nodes were excised by the harmonic scalpel, and the inferior thyroid veins were ligated. Thus, the right-side thyroid as well as the CCLN in Level VIA could be freed from the trachea.

**Fig. 3:**
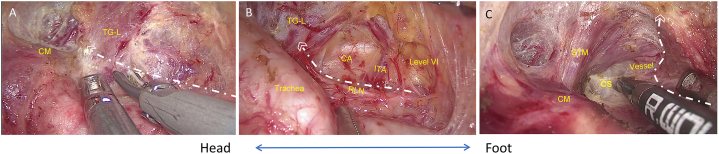
**Management of the left thyroid gland.** A) Dissection continued to the left side of the thyroid gland by dissecting the dorsal isthmus of the thyroid gland laterally to the tracheoesophageal groove of the left side. B) The left RLN was identified and traced at the tracheoesophageal groove, and the left thyroid gland and level VI lymph nodes were separated from the RLN. C) Superior vessels of the TG-L were ligated by exposing the cricothyroid space. Note: CM, cricothyroid muscle; TG-L, left thyroid gland; ITA, inferior thyroid artery; CA, carotid artery; CS, cricothyroid space; STM, sternothyroid muscle. White lines represent cutting lines.

**Fig. 4:**
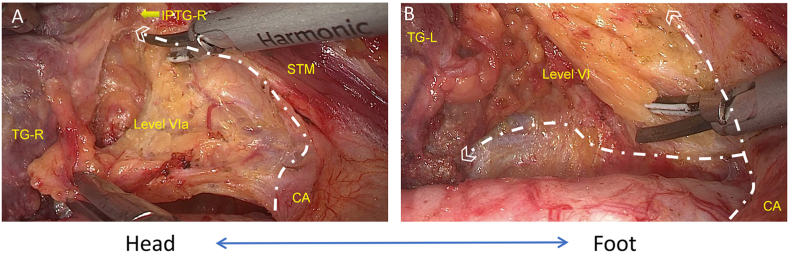
**Management of bilateral central compartment lymph nodes.** A) The lower boundary of Level VIa (right side) was dissected and freed from the muscle, and the thymus was preserved. B) Dissection was continued along the carotid artery, and the external and lower boundary of the left Level VI was dissected. Note: TG-R, right thyroid gland; IPTG-R, right inferior parathyroid gland; STM, sternothyroid muscle; TG-L, left thyroid gland; CA, carotid artery. White lines represent cutting lines.

**Fig. 5:**
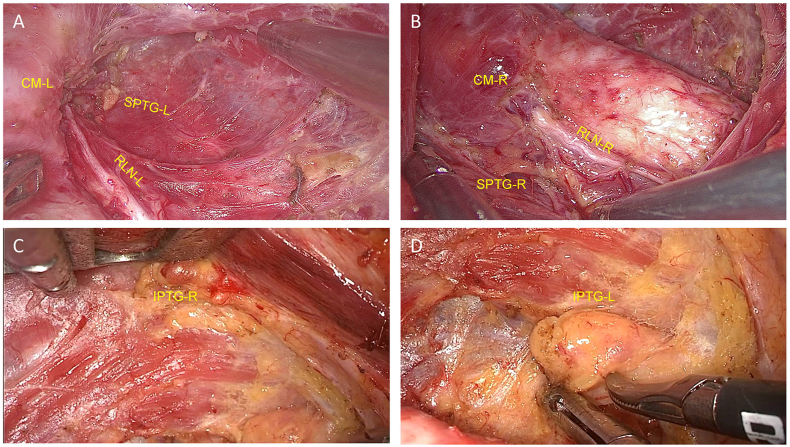
Single-incision, gasless, transaxillary endoscopic total thyroidectomy and bilateral central neck dissection were performed while the bilateral RLN and PTG were preserved *in situ*. Note: RLN, left recurrent laryngeal nerve; SPTG, superior parathyroid gland; CM, cricothyroid muscle; IPTG, inferior parathyroid gland.

To avoid additional skin incisions, the procedure continued to the left side of the thyroid by dissecting isthmus of the thyroid laterally to the tracheoesophageal groove. Adjusting the retractor by placing it under the freed thyroid gland (right lobe and isthmus), the contralateral gland and the CCLN were elevated (Fig. 3A). Then, the left RLN can be identified at the inferior pole of the thyroid (Fig. 3B). Forceps were used to apply medial traction to the trachea, resulting in the exposure and separation of the left RLN from both the left-side thyroid and the CCLN to each respective boundary: the superior boundary - RLN insertion point, external boundary - carotid artery, and lower boundary - level of innominate artery. The thyroid was moved laterosuperiorly, and dissection proceeded into the cricothyroid space. After individually ligating the superior thyroid vessels, the dorsal side of the thyroid was dissected close to the superior pole. Meanwhile, the left-side superior PTGs and EBSLN were preserved (Fig. 3C). During the dissection of the bilateral CCLN, the thymus became visible after exposing its inferior margin (Fig. 4A and B). In order to expose the STM, the retractor was adjusted, allowing for detachment of the bilateral thyroid and CCLN from the surrounding muscle, utilizing harmonic scalpels. Subsequently, a complete *en bloc* resection of the bilateral thyroid, CCLN, and the adjacent mesentery was performed (Fig. 5A–D). Finally, the specimen was carefully placed in a specimen bag, extracted from the incision, and taken away for further analysis. A surgical video comprising each step can be viewed in the supplementary files.

Supplementary data related to this article can be found online at https://doi.org/10.1016/j.heliyon.2024.e24802

The following are the Supplementary data related to this article:

Video Video

### 2.3 Outcomes of interest

The overall complication rate (for instance, temporary or permanent vocal cord paralysis and hypocalcemia, postoperative hemorrhage or hematoma, seroma, neck and chest wall paresthesia, difficulty swallowing, and etc.) was the primary endpoint of this study. Fiberoptic pharyngorhinoscopy was utilized to assess vocal cord function both prior to and one day after the surgical procedure. Vocal cord paralysis or hypocalcemia was considered temporary if recovery was observed within six months postoperatively. There were several secondary endpoints to be considered, including the mean operative time (min), blood loss during the surgery (mL), duration of hospital stay (days), number of retrieved lymph nodes, total medical cost (measured in USD), and cosmetic satisfaction (evaluated using the visual analog scale [VAS], ranging from 1 [unsatisfied] to 10 [satisfied]). Additionally, demographic information results (including sex, age, pathological results, and etc.) were documented. Follow-up assessments were conducted at one month, three months, six months, and one year following the surgery.

### 2.4 Statistical analysis

Categorical variables are expressed as frequencies and percentages. And, continuous variables are expressed as medians (interquartile ranges). Data were evaluated with SPSS software, version 22.0 (IBM, New York, USA).

## 3 Results

Overall, 22 patients underwent single-incision, gasless, transaxillary endoscopic TT + BCND. Table 1 presents the surgical findings, with the patients' median age at the time of the procedure being 36.5 years (25–47), and exclusively comprising female participants. The median size of the largest tumor encountered during the study was 0.6 (0.4–1) cm.

**Table 1:** Demographic data and characteristics of patients undergoing total thyroidectomy and bilateral central neck compartment dissection via single-incision, gasless, transaxillary endoscopic approach.

Variables	Number of patients, n = 22
**Sex**	
Male	0
Female	22
**Age, [years]**^a^	36.5(25–47)
**BMI, [kg/m**^**2**^**]**^a^	22.3(20.5–26.9)
**ASA score (%)**	
I	17(77.3)
II	4(18.2)
III	1(4.5)
IV	0
**Thyroid function (%)**	
Normal	19(86.4)
Hyperthyroidism	1(4.5)
Hypothyroidism	2(9.1)
**Largest nodule size**, **[cm]**^a^	0.6(0.4–1)

a Expressed as median (interquartile ranges).

Table 2 presents with the median (IQR) total surgical time was 143 (85–160) min. The median of subcutaneous dissection time for the creation of the working space was 23.5 (21–30) min. No instances of intraoperative bleeding (major vessel damage) or damage to the trachea or esophagus were observed. In all cases, the RLNs were identified and preserved. No patients experienced conversion to open surgery. There was 9.1 % (2) of patients with minor postoperative complications, including pectoral and/or cervical hypoesthesia, occurred and resolved spontaneously by the sixth month after surgery. Major complications occurred in two patients. One patient (4.5 %) experienced transient unilateral RLN palsy, one (4.5 %) experienced transient hypocalcemia, and no patient developed subcutaneous hematoma. All these complications resolved within 1 month after surgery. No postoperative bleeding, seroma formation or permanent complications were occurred. The median length of postoperative hospital stay was 4 (3–4.5) days (Table 2). The median (IQR) hospitalization expenses for these patients was 3848 (3781–4145) USD.

**Table 2:** Surgical and pathological outcomes.

Variables	Number of patients, n = 22
**Subcutaneous dissection**, **[min]**^a^	23.5(21–30)
**Total surgical time**, **[min]**^a^	143(85–160)
**Intraoperative bleeding (%)**	
≤10 mL	20(90.9)
>10 mL	2(9.1)
**Complications (%)**	
**Major**	
RLN paralysis (unilateral, transient)	1(4.5)
Hypocalcemia(transient)	1(4.5)
subcutaneous hematoma	0(0)
**Minor**	
pectoral hypoesthesia	1(4.5)
cervical hypoesthesia	1(4.5)
**Duration of hospital stay, [days]**^a^	4(3–4.5)
**Hospitalization expenses, [USD]**^a^	3848(3781–4145)
**Papillary carcinoma (%)**	
Classical	19(86.4 %)
Follicular variants	3(13.6 %)
Others	0(0)
**Microscopic extrathyroidal extension (%)**	7(31.8 %)
**Number of retrieved lymph nodes**^a^	10.5(8–15)
**Number of metastatic lymph nodes**^a^	1.05(0–5)

a Expressed as median (interquartile ranges).

Table 2 presents the postoperative pathological findings. Pathological reports validated bilateral PTMC diagnosis for all patients. Furthermore, the median (IQR) count of lymph nodes yielded was 10.5 (8–15). No patients underwent postoperative RAI. One year after surgery, the median Tg level was 0.14 ng/mL (<0.04–0.17) under TSH suppression therapy, while their TgAb levels were negative. All of the patients were satisfied with their postoperative cosmetic results.

## 4 Discussion

This study reported the technique of a single-incision, gasless, transaxillary endoscopic TT + BCND in patients with bilateral PTMC, as well as its early oncological outcomes. Previous studies from Korea and China have revealed that gasless unilateral axillary thyroidectomy (GUA) can be performed safely [8,11,12]; Nevertheless, numerous constraints hinder its extensive practicality, including the requirement for proficient surgical techniques in minimally invasive surgery (steep learning curve), and above all, the necessity for TT + BCND (challenges in contralateral side management) [4,13]. To achieve bilateral surgery with a transaxillary approach, surgeons have made another incision on the contralateral side or implemented the Da Vinci system. Only a few experiences of TT and ipsilateral CND (tumor side) by the GUA approach have been reported [6,7,14]. However, none of these studies addressed the technical difficulty of bilateral surgery through unilateral access since the contralateral thyroid and central neck lymph nodes was obstructed by the trachea.

The fundamental idea behind this procedure is to employ a retractor that enables the upward traction of the thyroid, the CCLN, and the muscle as a cohesive unit. This leads to the formation of a surgical plate at the posterior boundaries of the thyroid and the CCLN, so surgeons can perform surgeries from a posterior approach guided by the thyroid mesentery. It has several advantages, such as easier access to the dorsal portion of the thyroid gland (traditionally requiring one hand to retract the gland upward) and thus feasible bimanual endoscopic action, increased tension between tissues for better manipulation, and increased likelihood of total radical resection. We chose a right-sided incision for better resection of managed level VIb lymph nodes because the surgeon was right-handed. The difficult point of the technique lies in managing the contralateral thyroid and the CCLN. To address this issue, we initially separation the pretracheal space, the dorsal region of the left lobe was exposed. Since the RLN was anatomically close to the tracheoesophageal groove on the left side, the retractor was used to pull the thyroid as well as the CCLN harboring the left RLN upwardly. Then, forceps were used to expose the RLN, and dissecting the vessels vertical to the RLN. Furthermore, the CCLN on the left side was resected *en bloc* after the RLN was properly exposed and preserved. In the current study, the median number of retrieved lymph nodes in bilateral CND was 10.5, which is consistent with our previous study suggesting the optimal number (11) in an open bilateral CND [15,16]. Since the indication was very restricted (early stage), our preliminary result showed a satisfactory number of LNY in CND (no less than other endoscopic or open approaches), thus reflecting the thoroughness of this technique [8,12]. In addition, management of the contralateral gland, especially the insertion point of the RLN, is important. As we described above, we traced the RLN to its entry point by adjusting the angle of the optical fiber and retracting the trachea. We believe this management is acceptable considering the ATA guidelines, and Tg/TgAb and ultrasonographic images indicated no sign of recurrence at the 1-year postoperative follow-up.

There is no convert to open surgery occurred, and the total procedure length was comparable to that of other endoscopic approaches [[17], [18], [19]]. Moreover, the procedure offers a lateral surgical perspective, resembling the open approach and providing a convenient environment for the surgical team [20]. Only one patient (4.5 %) experienced temporary RLN palsy, which recovered within 1 month. We speculated that there was thermal damage caused by harmonic scalpels because all RLNs were exposed and monitored during the surgery. One patient experienced temporary hypocalcemia, and they recovered within 1 month. The main reason for this result is that it is difficult to preserve contralateral PTGs, especially inferior PTGs. To better expose the contralateral gland and the RLN, harmonic scalpels were used to dissect the inferior thyroid arteries at their proximal ends; therefore, the vessel branches into inferior PTGs could not be properly preserved. Thus, the magnified view in endoscopy offered us the chance to distinguish and preserved the vessels of the PTGs [21]. Some of the studies have shown that if the PTGs cannot be preserved *in situ*, autotransplantation is recommended for suspicious PTGs confirmed by an intraoperative frozen section and this procedure will not increase the complication rates of permanent hypocalcemia [22,23]. The major and minor complication rates were not higher than those in other reports [19], indicating the safety and feasibility of the modified approach.

Another advantage of our modified technique is that it allows complete bilateral surgery via unilateral access without bilateral axillary incisions or robotic system. It has been well illustrated that robotic systems can improve surgical precision by providing magnified three-dimensional images and highly maneuverable wristed instruments [24]. However, the medical cost of the robotic system is expensive, and the average cost of surgery in the US was $13,670 according to a previous report [25]. In the current study, on the other hand, the median medical cost for each patient was only $3848. In addition, most robotic systems are located in major cities; therefore, they are relatively unavailable for the remaining population. Our modified technique only requires a regular laparoscopic system and instruments. After all, the current technique has a tremendous advantage from the medical economic perspective.

However, the current study has several limitations. We limited the patient inclusion criteria to investigate the surgical results specifically in the subgroup of patients who could derive the maximum advantages from our approach: bilateral PTMC and a desire for scar-free in the neck. However, selection bias may have affected the results and should not be neglected. Only 22 PTMC patients were included which is relatively small. Since this technique was recently modified, this preliminary study was conducted and reported its technique skills as well as surgical metrics. The potential advantages of this technique over traditional approaches, such as alleviated postoperative pain, improved quality of life after surgery were not investigated in the current study. In the future, a prospective, randomized cohort study (NCT06051838) will be conducted to evaluate the feasibility of treatment for bilateral PTMC with other approaches (open surgery/other endoscopic approaches). In addition, this method reduced the impact of scars by hiding the cut in the axilla, which was enveloped in the patient's armpit; notwithstanding, the use of a 5-mm trocar in this strategy still results in a skin scar located underneath the incision. For this reason, the transoral technique, including the periosteal, sublingual oral vestibular approach (TOETVA), was developed to achieve excellent cosmetic results [26]. The TOETVA technique has attracted international attention because of its advantages of providing an operative view and workspace for TT + BCND [27,28]. Nonetheless, the controversy surrounding the efficacy of TOETVA persists when juxtaposed with GUA. This stems from the inception of an inverted visual anatomical perspective and the cramped surgical setting, where endoscopic tools are in such close proximity [29]. Some researchers have revealed that the GUA approach has advantages in shorter surgical length, and less paresthesia over TOETVA [30]. In the current study, all patients were satisfied with the cosmetic results, indicating that our modified technique is acceptable for selected patients. Third, the patients enrolled did not receive RAI therapy, however, the postoperative suppressive Tg level and retrieved lymph nodes in the central neck were satisfactory.

## 5 Conclusion

The current study suggested that single-incision, gasless, transaxillary endoscopic TT + BCND could be a secure alternative for managing bilateral PTMC.

## Ethics statement

The study was approved by the Ethics Committees of Nanfang Hospital with the number: NFEC-2021-324, and all patients were fully informed and provided written consent.

## Funding statement

This work was supported by the 10.13039/501100001809National Natural Science Foundation of China (No. 82203778 and 82373366), Guangdong Basic and Applied Basic Research Foundation (No. 2022A1515010621), Science and Technology Program of Guangzhou (No. 2024A04J9949), Clinical Research Funding of 10.13039/501100010112Nanfang Hospital (2021CR017), and Featured Clinical Technique of Guangzhou (2023P-TS02).

## Data availability statement

Data will be made available on request.

## CRediT authorship contribution statement

**Shi-Tong Yu:** Writing – original draft, Conceptualization, Formal analysis, Project administration, Writing – review & editing. **Jun-Na Ge:** Investigation, Data curation, Resources, Visualization. **Bai-Hui Sun:** Investigation, Software, Validation. **Zhi-Gang Wei:** Visualization, Writing – review & editing. **Zhi-Cheng Zhang:** Methodology, Software. **Wei-Sheng Chen:** Data curation, Visualization. **Ting-Ting Li:** Data curation, Formal analysis. **Shang-Tong Lei:** Supervision, Funding acquisition, Conceptualization, Methodology.

## Declaration of competing interest

The authors declare that they have no known competing financial interests or personal relationships that could have appeared to influence the work reported in this paper.
